# Intra- and inter-species interactions within biofilms of important foodborne bacterial pathogens

**DOI:** 10.3389/fmicb.2015.00841

**Published:** 2015-08-20

**Authors:** Efstathios Giaouris, Even Heir, Mickaël Desvaux, Michel Hébraud, Trond Møretrø, Solveig Langsrud, Agapi Doulgeraki, George-John Nychas, Miroslava Kačániová, Katarzyna Czaczyk, Hülya Ölmez, Manuel Simões

**Affiliations:** ^1^Department of Food Science and Nutrition, Faculty of the Environment, University of the Aegean, Myrina, Lemnos Island, Greece; ^2^Nofima, Norwegian Institute of Food, Fisheries and Aquaculture Research, Ås, Norway; ^3^INRA, UR454 Microbiologie, Centre Auvergne-Rhône-Alpes, Saint-Genès-Champanelle, France; ^4^Laboratory of Microbiology and Biotechnology of Foods, Department of Food Science and Human Nutrition, Faculty of Foods, Biotechnology and Development, Agricultural University of Athens, Athens, Greece; ^5^Department of Microbiology, Faculty of Biotechnology and Food Sciences, Slovak University of Agriculture in Nitra, Nitra, Slovakia; ^6^Department of Biotechnology and Food Microbiology, Poznan University of Life Sciences, Poznań, Poland; ^7^TÜBİTAK Marmara Research Center, Food Institute, Gebze, Kocaeli, Turkey; ^8^Laboratory for Process Engineering, Environment, Biotechnology and Energy, Department of Chemical Engineering, Faculty of Engineering, University of Porto, Porto, Portugal

**Keywords:** biofilms, foodborne pathogenic bacteria, interactions, aggregation, competition, cooperation, communication

## Abstract

A community-based sessile life style is the normal mode of growth and survival for many bacterial species. Under such conditions, cell-to-cell interactions are inevitable and ultimately lead to the establishment of dense, complex and highly structured biofilm populations encapsulated in a self-produced extracellular matrix and capable of coordinated and collective behavior. Remarkably, in food processing environments, a variety of different bacteria may attach to surfaces, survive, grow, and form biofilms. *Salmonella enterica, Listeria monocytogenes, Escherichia coli*, and *Staphylococcus aureus* are important bacterial pathogens commonly implicated in outbreaks of foodborne diseases, while all are known to be able to create biofilms on both abiotic and biotic surfaces. Particularly challenging is the attempt to understand the complexity of inter-bacterial interactions that can be encountered in such unwanted consortia, such as competitive and cooperative ones, together with their impact on the final outcome of these communities (e.g., maturation, physiology, antimicrobial resistance, virulence, dispersal). In this review, up-to-date data on both the intra- and inter-species interactions encountered in biofilms of these pathogens are presented. A better understanding of these interactions, both at molecular and biophysical levels, could lead to novel intervention strategies for controlling pathogenic biofilm formation in food processing environments and thus improve food safety.

## Introduction

For many years it was believed that microorganisms inhabit the planet mainly in a planktonic form, as free-living cells, but it is now widely accepted that most of them reside primarily in biofilms. These are assemblages of microorganisms adherent to each other and/or to a surface and embedded in a scaffold of self-produced extracellular polymeric substances (EPS; Stoodley et al., 2002; Hall-Stoodley et al., 2004). The last decades, biofilm formation by bacterial pathogens has attracted much attention, mainly in the medical and food processing fields, due to its potential risks, including antimicrobial resistance, persistence, and virulence factor production (Stewart and Costerton, 2001; Chmielewski and Frank, 2003; Jahid and Ha, 2014; Bridier et al., 2015). For simplicity, most of the research on biofilms has taken a reductionist approach, where single species biofilms have been extensively investigated. However, biofilms in nature mostly comprise multiple species, where inter-species interactions can shape the development, structure and function of these communities (Yang et al., 2011b; Elias and Banin, 2012; Rendueles and Ghigo, 2012; Burmølle et al., 2014). Therefore, in recent years there has gradually been a shift in focus toward examining the complexity and interactions in multi-species biofilms. The use of high throughput and high resolution methods has facilitated this development in revealing complex microbial interactions including genetic, metabolite exchange and signaling to occur between microorganisms in biofilm communities (Møller et al., 1998; Madsen et al., 2012; McLean and Kakirde, 2013). Such interactions may influence growth and survival of biofilm community members and also their potential virulence properties which could on their turn influence the overall pathogenicity of such structures (Peters et al., 2012a).

Living in biofilms allows bacteria to interact with each other and function as a group for coordinated activities (Nadell et al., 2009). These cell-to-cell interactions influence both the temporal and spatial formation of a highly organized community architecture and are roughly categorized as either cooperative or competitive (James et al., 1995; Shirtliff et al., 2002; Wuertz et al., 2004; Moons et al., 2009). Their significance was first realized and thoroughly described for bacteria residing in the oral cavity (Kolenbrander et al., 2006), while equivalent patterns were later revealed in biofilm bacteria isolated from various non-host environments, including artificial habitats, such as food processing. As an example, dental plaque is a well-recognized biofilm community, which is characterized by its broad biodiversity (>700 species) and high cell density (10^11^ cells/g wet wt), with the intra- and inter-species interactions encountered inside this to be well described (Kuramitsu et al., 2007; Hojo et al., 2009; Huang et al., 2011; Diaz, 2012; Guo et al., 2014). For instance, bacterial coaggregation is a main type of cooperative interactions encountered among oral bacteria that facilitate coadhesion of bacterial pairs to the tooth surface (Rickard et al., 2003). In terms of coaggregation, the succession of bacterial biofilms is tightly controlled by specific cell surface-associated receptor-ligand interactions and this often results in enhanced levels of multi-species biofilm formation. Thus, some attached pioneering bacteria will be recognized and serve as anchor for secondary colonizers, while the close contact resulting from coaggregation events facilitates cooperation between the different species.

In a true cooperative partnership, all species profit in some way from the presence of others, leading to an enhanced overall fitness of the biofilm consortium. This can be achieved through the provision of biofilm formation capacity to the community, by producing substances that can serve as nutrients for the cohabiting species, by removing metabolites that would otherwise slow down growth, or by any combination of all these. Bacteria can also collaborate to degrade compounds, which often results in a three dimensional organization whereby structures are formed that facilitate the transfer of the primary degradation product toward secondary degraders that are usually clustered around the primary degrader. Cooperative metabolic interactions are also suggested by the observation that certain bacterial species can modify the local microenvironment, making it more suitable for the growth of other organisms, for instance by changing the pH or concentration of oxygen. Thus, organisms which are able to metabolize oxygen could favor the growth of nearby anaerobic organisms (Stewart and Franklin, 2008).

Common competitive interactions are antagonism for limiting nutrient sources, oxygen and available space to colonize. One important factor in determining the bacterial composition of a biofilm is clearly the availability of nutrients with the competition for nutrients and other growth parameters to certainly be an important driving force for the development of biofilm structure. Thus, numerous experimental data obtained in the laboratory show how different microorganisms may effectively outcompete others as a result of better utilization of a given energy source (Wuertz et al., 2004). Competition may also be done through the production of compounds (e.g., bacteriocins, organic acids, biosurfactants, enzymes) that may inactivate, inhibit the growth of, or prevent attachment of other species or even provoke detachment of their cells from the biofilm structures (Rendueles and Ghigo, 2012).

It is well accepted that bacteria in biofilms are more protected against various stresses than planktonic exponentially growing cells (Anderson and O’Toole, 2008; Coenye, 2010). Several studies have further suggested that interactions between biofilm bacteria could influence their relative resistance (Burmølle et al., 2006; Simões et al., 2009; Uhlich et al., 2010; van der Veen and Abee, 2011; Giaouris et al., 2013; Lee et al., 2014b). In the presence of toxic compounds, the relative abundance of the different species in a population shifts toward the species best equipped to deal with the compound. However, sensitive strains may profit from protection by more resistant populations. The most straightforward case is when the resistant species completely detoxifies the compound. In other cases, the sensitive strain is protected through physical shielding by the resistant strain. Interestingly, advanced imaging techniques have identified that microorganisms are clustered within biofilms and are not randomly distributed (Neu et al., 2010). Such structural organization and architectural differentiation has been shown to enhance persistence of a synthetic biofilm consortium (Brenner and Arnold, 2011). Another mechanism that might lead to higher resistance is the interaction between the matrix polymers of the different species. Thus, even when neither of the two partners is intrinsically resistant to the treatment, enhanced resistance can still occur (Moons et al., 2009).

Increased cell density also favors chemical cell-to-cell signals involved in social interactions in biofilms (Kjelleberg and Molin, 2002; Li and Tian, 2012). Thus, many bacteria are known to regulate their cooperative activities and physiological processes through a mechanism called quorum sensing (QS), in which bacterial cells communicate by producing, detecting, and responding to small diffusible signal molecules called autoinducers. Davies et al. (1998) was the first to show the involvement of cell-to-cell signals in biofilm development by describing their role in biofilm formation by the common opportunistic pathogen *Pseudomonas aeruginosa*. Nowadays, it is widely accepted that bacteria can form well-organized sessile communities and communicate for coordinated activities or social life that was once believed to be restricted to multicellular organisms (Webb et al., 2003; Nadell et al., 2009; Mitri et al., 2011). The close proximity of cells in biofilms, the spatio-chemical conditions enabling bacterial coexistence and the compound-retaining matrix provide optimal conditions for QS-mediated gene regulation. As a consequence, increasing evidence shows that QS is an integral component of bacterial global gene regulatory networks responsible for bacterial communication in biofilms (Irie and Parsek, 2008). However, research on the functional consequences of QS in biofilms and more importantly in multi-species ones remains in its infancy.

Another interaction which can have major consequences for the physiology of biofilms, as well as evolutionary outcomes, is the genetic exchange between biofilm residents, with these communities to be uniquely suited for horizontal gene transfer (HGT; Molin and Tolker-Nielsen, 2003; Madsen et al., 2012). HGT through conjugation occurs in multi-species biofilms and in contrast, to mono-species biofilms, this potentially results in new genetic combinations and facilitate the creation of novel genotypes that could become problematic for humans (Christensen et al., 1998). Various conjugative plasmids, such as those encoding adhesive structures (such as fimbriae), have been characterized, with their presence to strongly induce biofilm formation by their hosts (Ghigo, 2001; Reisner et al., 2006; Burmølle et al., 2008). Multi-species biofilms also facilitate HGT by transformation. The large amounts of extracellular DNA (eDNA) in biofilms is likely to be an important common source of usable genetic information for members of the biofilm community (Das et al., 2013b).

From all the above it is clear that microbial cells in biofilms physically interact and maintain close relationships, which have led to the smart perception of biofilms as cities of microorganisms (Watnick and Kolter, 2000). Microbial diversity in these communities leads to a variety of complex relationships, involving both inter- and intra-species interactions (Moons et al., 2009). Certainly, microbial community stability can be achieved only when a natural balance is established among different microorganisms within the same biological niche, and this balance is often the result of the constant “war and peace” activities experienced by all the members of the community. The purpose of this article is to review the intra- and inter-species interactions shown to be encountered in biofilms of the following common foodborne bacterial pathogens: *Salmonella enterica, Listeria monocytogenes, Escherichia coli*, and *Staphylococcus aureus* and try to summarize—where possible—their underlying mechanisms, as well as their impact on the physiology and function of these communities. Many of these intercellular interactions are governed by cell surface structures (adhesins) and exopolymers that these pathogens carry/produce and are mainly responsible for their ability to bind to different surfaces (including cells) and/or compounds. However, for detailed information on these molecules (involved in primary adhesion, including their receptors), reader is referred to the recently published review article of Jaglic et al. (2014). In the present review, only those of these structures that are also known to be involved in biofilm development, mostly by resulting in coaggregation of cells (of the same and/or different species), will be mentioned (and not all the surface adhesins in general). Given the huge importance of cell-to-cell interactions in the establishment, maintenance, and function of biofilm communities, a better understanding of the mechanisms by which these bacteria interact in a biofilm should help to develop strategies for their elimination at source.

## *Salmonella* spp.

*Salmonella enterica* is one of the most significant enteric foodborne bacterial pathogens (Ruby et al., 2012), with the non-typhoidal strains to be classified into more than 2500 serovars of which the serovars Typhimurium and Enteritidis are the most prevalent (Foley et al., 2013). These bacteria are well known to attach to various biotic and abiotic surfaces, such as those of plants, the eukaryotic host, industrial facilities, and medical supplies, and create biofilms (Steenackers et al., 2012).

### Intraspecies Interactions

The multicellular behavior of bacteria has been the subject of much recent interest (Dunny et al., 2008). In addition, the structural and physiological complexity of biofilms has led to the idea that they are coordinated and cooperative groups, analogous to multicellular organisms (Nadell et al., 2009). Regarding *Salmonella* spp., rdar is a multicellular morphotype which biofilm forming strains present when these are cultured on Congo red agar plates, due to the red, dry and rough appearance of the colonies. This is characterized by the co-expression of the extracellular matrix components curli (thin aggregative) fimbriae (Tafi or SEF17 fimbriae) and cellulose (Zogaj et al., 2001; Römling, 2005). Curli were first discovered in the late 1980s on *E. coli* strains that caused bovine mastitis and these are mainly involved in adhesion to surfaces, cell aggregation, and biofilm formation (Austin et al., 1998). Curli also mediate host cell adhesion and invasion, and they are potent inducers of the host inflammatory response (Barnhart and Chapman, 2006). Isolates of *Salmonella* spp. deficient in curli and/or cellulose production have been found to be least effective in biofilm formation (Solomon et al., 2005). In agreement, Jain and Chen (2007) demonstrated that curli impart attachment ability to *Salmonella* spp. and, upon co-expression with cellulose, enhance biofilm formation on certain abiotic surfaces. However, Castelijn et al. (2012) demonstrated that these two polymers contribute specifically to biofilm production under low nutrient conditions at ambient temperatures and that other unknown components are conceivably more important during biofilm formation at 37°C and/or in nutrient-rich conditions.

In *Salmonella* spp., the expression of curli and cellulose is dependent on the transcriptional regulator CsgD, whose expression integrates many environmental signals, such as starvation, oxygen tension, temperature, pH, and osmolarity (Gerstel and Römling, 2003). CsgD positively regulates the transcription of the *csgBAC* operon, which encodes the structural subunits for curli (Hammar et al., 1995), and contributes indirectly to cellulose production by activating the transcription of *adrA* (Gerstel and Römling, 2003). Two operons, *bcsABZD* and *bcsEFG*, are required for cellulose biosynthesis (Solano et al., 2002). AdrA is a diguanylate cyclase that synthesizes the second messenger signaling molecule bis-(3′-5′)-cyclic dimeric guanosine monophosphate (cyclic-di-GMP), the effector molecule that binds to and allosterically activates cellulose synthase (Simm et al., 2007). c-di-GMP is widespread throughout the bacterial domain and plays a vital role in regulating the transition between the motile planktonic lifestyle and the sessile biofilm forming state (Le Guyon et al., 2015). The GGDEF and EAL domain-containing proteins, acting as phosphodiesterases, are involved in turnover of this secondary messenger and play a determinative role in the expression level of multicellular behavior in *Salmonella* Typhimurium (Simm et al., 2007).

**FIGURE 1:**
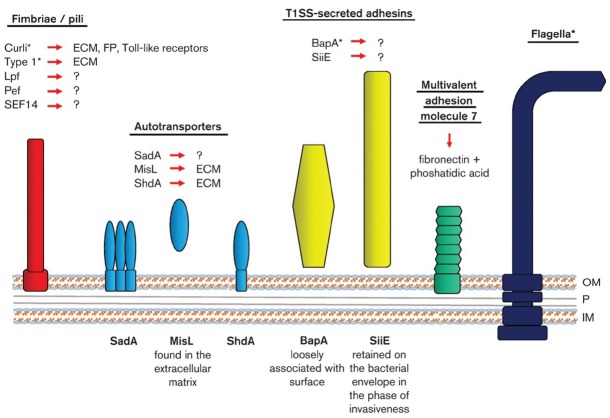
**Schematic drawing of the cell envelope of ***S. enterica*** (OM, outer membrane; P, periplasm with peptidoglycan; IM, inner membrane) with symbolized bacterial adhesion molecules including their receptors (?, unknown receptors; ECM, extracellular matrix proteins; FP, fibrinolytic proteins).** *Mediates adhesion to abiotic surfaces and biofilm formation. The structures depicted do not reflect the real macromolecule structures. Figure obtained after permission from Jaglic et al. (2014), Copyright Society of General Microbiology© 2015.

Besides curli, depending on the serotype, gene clusters for more than 10 different fimbrial adhesins have been identified, such as plasmid encoded fimbriae (Pef) and long polar fimbriae (Lpf). In addition, autotransporter adhesins (e.g., ShdA, MisL, and SadA) and type I secreted large repetitive adhesins (e.g., SiiE and BapA) are known (Wagner and Hensel, 2011). Although the functions of many of these adhesins, as well as of putative others (such as flagella, capsular polysaccharides, lipopolysaccharides) are not always very well understood, several studies have revealed their putative roles in cell aggregation, multicellular behavior, and biofilm formation (Römling and Rohde, 1999; Prouty et al., 2002; White et al., 2003; Ledeboer and Jones, 2005; Anriany et al., 2006; Jonas et al., 2007; Crawford et al., 2008). For instance, SadA, is a trimeric autotransporter adhesin (TAA) of *S.* Typhimurium which belongs to type V secreted proteins and its expression results in cell aggregation, biofilm formation, and also increased adhesion to human intestinal Caco-2 epithelial cells (Raghunathan et al., 2011). BapA is a large cell-surface protein required for biofilm formation by *Salmonella* Enteritidis and this is secreted through a type I protein secretion system (BapBCD) situated downstream of the *bapA* (Latasa et al., 2005). Interestingly, the expression of *bapA* is coordinated with that of genes encoding curli fimbriae and cellulose, through the action of *csgD*. *S.* Typhimurium requires the Lpf, Pef, and Tafi fimbriae for biofilm formation on HEp-2 tissue culture cells and chicken intestinal epithelium (Ledeboer et al., 2006), while the contact of these bacteria with cultured epithelial cells has also been shown to result in the formation of unusually wide tubular appendages attaching bacteria to the epithelial cells (Reed et al., 1998). In addition, these adhesive appendages have been shown to interconnect bacteria in biofilms grown on gallstones or coverslips (Galkina et al., 2011). Type 3 fimbriae, encoded by the conjugative plasmid pOLA52, enhance biofilm formation and transfer frequencies in *Enterobacteriaceae* strains (Burmølle et al., 2008). *S.* Enteritidis enteropathogens also produce a variety of potentially adherent fimbrial types, including SEF14, SEF18, and SEF21 (type I; Austin et al., 1998). Figure 1 shows a schematic overview of the most known *Salmonella* adhesion molecules including their receptors.

Pathogenicity islands typically accommodate large clusters of genes that contribute to a particular virulence phenotype, with *S.* Typhimurium possessing at least five such pathogenicity islands (SPIs; Marcus et al., 2000). Among these, SPI1 is primarily required for bacterial penetration of the epithelial cells of the intestine. Interestingly, Jennings et al. (2012) observed that *S.* Typhimurium cultures containing cloned SPI1 secretion system displayed an adherent biofilm and cell clumps in the media. This bacterial aggregation phenotype was associated with hyper-expression of SPI-1 type III secretion functions. With respect to mobile genetic elements, *Salmonella* genomic island 1 (SGI1) is associated with the multiple-drug-resistance (MDR) of *S.* Typhimurium DT104 strain (Boyd et al., 2001), while Malcova et al. (2008) has also demonstrated the additional positive effect of SGI1 on biofilm formation. In accordance, in recent years some other studies have revealed the role of multidrug efflux pumps in the ability of *Salmonella* spp. to produce biofilm (Baugh et al., 2012). Thus, resistance, biofilm production and fitness seem to be interrelated (Fàbrega et al., 2014). Noteworthily, eDNA has been found to inhibit biofilm development by *S.* Typhimurium and *S.* Typhi on abiotic surfaces (Wang et al., 2014).

### Interspecies Interactions

Several studies have reported that biofilm production of *Salmonella* spp. may be promoted by the presence of other bacteria. In dual-species biofilms on stainless steel in drip-flow reactors, *Staphylococcus piscifermentans* and *Pseudomonas* sp. isolated from the feed industry increased biofilm growth of *S.* Agona compared to what was found in single species biofilms, with about threefold increases of the biovolume (Habimana et al., 2010b). In another flow system, with drinking water with acetate, *S.* Typhimurium did only form very thin-layered biofilm in monoculture on silicone tubes, but formed larger microcolonies when cultured together with a mixture of three strains of *Paenibacillus* sp., one *Bacillus* sp. and one strain of *Enterococcus* (Schaefer et al., 2013). The mechanism behind increased biofilm production was not investigated in these studies. However, in another study with a laboratory reactor simulating flow in water pipes, a much higher proportion of metabolically active cells were found in mixed biofilms than in single species biofilms. Also, the level of attachment of *S.* Enteritidis was seven times higher after 72 h in a dual biofilm with *Klebsiella pneumoniae* than in a single species biofilm (Jones and Bradshaw, 1997). Curli are important for biofilm formation of *Salmonella*. In a study of *E. coli* and *S.* Typhimurium, it was found that biofilm negative *Salmonella* could utilize parts of curli from *E. coli* and form biofilm on agar and pellicles (Zhou et al., 2012). In mixed biofilms of *S.* Typhimurium and *E. coli* O157:H7 on microtiter plates, *Salmonella* were generally found in higher numbers than *E. coli*, and a curli and cellulose producing *Salmonella* competed better with *E. coli* than a curli/cellulose negative *Salmonella* (Wang et al., 2013). *Salmonella* spp. or *E. coli* strains with negative EPS expression obtained significantly enhanced resistance to sanitation with a quaternary ammonium compound or chlorine by forming mixed biofilms with an EPS-producing companion strain of the other species (Wang et al., 2013).

A number of studies have demonstrated that the presence of other species may inhibit biofilm production of *Salmonella* spp. *S. aureus* dominated (99%) over *S.* Typhimurium in a constant-depth film fermentor (Knowles et al., 2005). *Salmonella* was primarily present in the top 40 μm of the biofilm and was not detected below 180 μm. This observation can be explained by that, in contrast to *S. aureus, Salmonella* require oxygen to produce biofilm and often form biofilm at liquid-air interphases (Scher et al., 2005). However, in another study where *E. coli* outcompeted *S. enterica* and *L. monocytogenes* in biofilms on different materials, *S. enterica* and *L. monocytogenes* were found close to the material surface, while *E. coli* was found in the biofilm top layer. The higher growth rate and exopolymer production ability of *E. coli* probably led this microorganism to outcompete the other two (Almeida et al., 2011). In a mixed species biofilm of *S. simulans, Lactobacillus fermentum, Pseudomonas putida, L. monocytogenes,* and *S.* Enteriditis, *P. putida* composed 98% of the population, while biofilm of *S.* Enteritidis was reduced by 1.5 log compared to the density of its population when this was grown as monoculture biofilm (Chorianopoulos et al., 2008).

Interbacterial communication signals like boronated-diester molecules (AI-2) and *N*-acyl homoserine lactones (AHLs) may be present in multi-species biofilms. *Salmonella* spp. can produce and respond to AI-2 and transcriptomics indicated an effect of AI-2 on biofilm formation in *S.* Typhimurium (Jesudhasan et al., 2010). However, Yoon and Sofos (2008a) found no differences in biofilm formation between AI-2 producing and non-producing *S.* Thompson. Brominated furanones which are believed to block QS signals have been shown to inhibit biofilm formation by *Salmonella* spp. (Janssens et al., 2008; Vestby et al., 2010, 2014). In the same way, grapefruit juice and its furocoumarins have been found to inhibit biofilm formation by *E. coli* O157:H7, *S.* Typhimurium and *P. aeruginosa* (Girennavar et al., 2008). These results suggest that targeting of microbial cell signaling processes could serve as a source to develop bacterial intervention strategies. Several types of bacteria have been reported to secrete compounds that inhibit biofilm of *Salmonella* spp. Heat stable compounds present in *Hafnia alvei* culture supernatant inhibited the early stage of biofilm development by *S.* Enteritidis on stainless steel. Although AHLs were detected in the supernatant, the role of AHLs could not be confirmed as synthetic AHLs did not affect the initial stage of biofilm formation by this pathogen (Chorianopoulos et al., 2010). The marine bacterium *Pseudoalteromonas* strongly inhibited adhesion of *Salmonella* spp. to glass through secretion of a compound of unknown identity (Dheilly et al., 2010). A probiotic *Lb. plantarum* strain led to 2-log decrease in biofilm forming colonies of *Salmonella* spp., compared to growing *Salmonella* spp. in monoculture. The effect was probably due to an unknown compound excreted by *Lb. plantarum* that also killed planktonic *Salmonella* spp. (Das et al., 2013a). Likewise, Woo and Ahn (2013) suggested that probiotic strains can be used as an alternative strategy to effectively reduce the biofilm formation in pathogenic bacteria through competition, exclusion, and displacement.

It has also been reported that *Salmonella* spp. can have an effect on biofilm of other bacteria. Thus, *S.* Typhimurium was found to outgrow and displace *E. coli* when it formed a biofilm on HEp-2 cells in a flow-through continuous culture system (Esteves et al., 2005). In biofilms on stainless steel, *S. enterica* influenced the intraspecies distribution of three *L. monocytogenes* strains in multi-species biofilms (Kostaki et al., 2012). The mechanism behind this interaction was not revealed. Most biofilm studies are on mixed cultures of bacteria, but two studies have also demonstrated interactions between *Salmonella* spp. and fungi. Tampakakis et al. (2009) showed that *S.* Typhimurium was able to secrete a heat stable substance that inhibited filamentation and biofilm formation of *Candida albicans* on silicone pads. In another study, *S.* Typhimurium was reported to rapidly attach to and forms biofilms on the hyphae of the fungus, *Aspergillus niger*. Interactions between cellulose produced by *S.* Typhimurium and chitin of *A. niger* was required for the production of the mixed biofilms (Brandl et al., 2011).

### Intercellular Interactions in Biofilms of *Salmonella*: Current Knowledge and Concepts for Future Research

The persistence of *Salmonella* within the food chain is a major health concern, with its ability to form biofilms in food processing environments to serve as a potential reservoir for the contamination of food products. Undoubtedly, this ability contributes to its survival in non-host environments, stress hardening and its transmission to new hosts (Giaouris and Nesse, 2015). Like other bacterial pathogens, *Salmonella* is able to produce (depending on the surrounding conditions) various cell surface structures (especially of proteinaceous and carbohydrate nature) that all may result in the efficient coaggregation of its own cells with each other, as well as with cells of other species, facilitating thereby the formation of either mono- or multi-species biofilm communities. For instance, the co-expression of two of these components, curli fimbriae and cellulose, was shown to lead in the formation of a highly hydrophobic network with tightly packed cells aligned in parallel in a rigid matrix (Zogaj et al., 2001). Interestingly, White et al. (2010) showed by comparing extracellular matrix-embedded, wild-type *S.* Typhimurium and the matrix-deficient *csgD* mutant that the two populations present distinct metabolite and gene expression patterns, with wild-type cells expressing genes mainly involved in gluconeogenesis and stress-resistance pathways. Noteworthily, the effect of the simultaneous presence of other bacteria on the ability of *Salmonella* to form biofilms seems to greatly vary depending on the environmental conditions and bacteria tested. Thus, in laboratory studies, *Salmonella* spp. may be inhibited, promoted, or apparently do not respond to the presence of other bacteria in a biofilm. However, to the best of our knowledge, nothing is yet known on whether these multi-species communities could influence the pathogenicity of *Salmonella* (possibly by up-regulating the expression of virulence genes), while very little is still known on the effect of QS on *Salmonella* biofilm formation, especially in multi-species environments (Boyen et al., 2009). Targeting the QS mechanisms may provide a promising strategy for combatting biofilms and their associated problems.

## Listeria monocytogenes

*Listeria monocytogenes* is a Gram-positive opportunistic and facultative intracellular bacterial pathogen that has served as a model organism for many virulence and biofilm related studies (Chavant et al., 2002; Møretrø and Langsrud, 2004; Cossart, 2011; Renier et al., 2011; Huang et al., 2012; da Silva and De Martinis, 2013). This is one of the major concerns in the food industry given that it can survive and even multiply in the harsh environmental conditions that exist in the production, processing, and storage of food products (Gandhi and Chikindas, 2007; Carpentier and Cerf, 2011). In addition, many *L. monocytogenes* strains can attach to various surfaces and form biofilms. However, strain and serotype differences occur, while conflicting results have been reported regarding correlations between biofilm forming abilities and persistence or phylogeny/lineage (Norwood and Gilmour, 2001; Djordjevic et al., 2002; Borucki et al., 2003; Di Bonaventura et al., 2008; Kadam et al., 2013; Valderrama and Cutter, 2013).

### Intraspecies Interactions

Concerning *L. monocytogenes* intracellular interactions, the involvement of PrfA, the transcriptional activator of virulence operons, in biofilm formation was initially reported by Lemon et al. (2010) and Zhou et al. (2011), while Travier et al. (2013) subsequently demonstrated that *actA*, one the PrfA-regulated genes, was responsible for this function. In fact, ActA mediates interactions between *L. monocytogenes* cells via direct ActA-ActA interactions resulting in bacterial aggregation and biofilm formation. This aggregation property of ActA favors long-term gut colonization and fecal shedding, playing a key role in persistence within the host and in transmission from the host back to the environment (Travier et al., 2013; Travier and Lecuit, 2014).

Another protein involved in cell-to-cell interactions in this bacterium is SecA2, which is a paralog of SecA, a peripheral ATPase essential for the passage of pre-proteins through the cytoplasmic membrane. The *secA2* gene has been identified in several pathogenic Gram-positive bacteria such as *L. monocytogenes* in which its deletion results in cell morphotype changes from discrete cells forming smooth colonies in wild type strain to long-chain cells forming rough colonies (Lenz and Portnoy, 2002). This colony morphotype is also observed when *cwhA* and *murA*, two genes encoding extracellular cell wall hydrolases, are deleted (Lenz et al., 2003; Renier et al., 2013, 2014) or when the SecA2-dependent secretion of their gene products is reduced (Machata et al., 2005). It is noticeable that this morphotype has been already observed from clinical patients, food samples and environmental biofilms (Rowan et al., 2000; Monk et al., 2004). In fact, this morphotypic conversion of bacterial cells is reversible and may have strong consequences on the ability of *L. monocytogenes* to colonize surfaces. In liquid medium, the inactivation of the SecA2 pathway results in extensive cell aggregation and sedimentation (Renier et al., 2014). So, the morphotypic conversion provides a significant advantage in listerial surface colonization under environmental conditions (Monk et al., 2004). It could be considered as an important risk factor for food processing plants and food products contamination but also of potential significance for asymptomatic human or animal carriage. Moreover, considering that biofilms are generally multi-species rather than mono-species, this cell differentiation could have consequences on *L. monocytogenes* implantation and interaction with other microbial species in various ecological niches (Sasahara and Zottola, 1993).

*L. monocytogenes* is a mobile bacterium thanks to 4–6 peritrichous flagella composed of thousands of monomers of the FlaA protein. However, this mobility is temperature-dependent because the transcription of *flaA* is stopped above 30°C. While flagella are important for biofilm formation of numerous bacterial species, their involvement in *L. monocytogenes* biofilm formation is quite controversial and seems dependent of environmental factors, namely more particularly growth medium, pH and temperature (Tresse et al., 2006, 2009), dynamic or static conditions (Rieu et al., 2008a). Undoubtedly, mutagenesis approaches have revealed that flagella and their motility play a role in *L. monocytogenes* biofilm formation. (Chang et al., 2012; Ouyang et al., 2012). However, this role seems to be limited to the positive role afforded by motility on the initial steps of surface attachment, probably by increasing the likelihood of encountering a surface and overcoming the repelling electrostatic forces, and not by flagella acting as surface adhesins *per se* (Vatanyoopaisarn et al., 2000; Lemon et al., 2007; Kumar et al., 2009; Jaglic et al., 2014). On the contrary, both non-flagellated and non-motile *L. monocytogenes* mutants were impaired in initial attachment but subsequently were revealed as hyper-biofilm formers when grown in flow cells (Todhanakasem and Young, 2008).

Contrary to the extracellular matrixes of many microbial biofilms, the one of *L. monocytogenes* seems to lack exopolysaccharides (Renier et al., 2011). Some observations by electron microscopy have sometimes revealed the presence of putative fimbriae-like structures binding cells to each other or to the surface (Hefford et al., 2005; Zameer et al., 2010; Renier et al., 2011). However, it was suggested that these fibrils resulted from the complete dehydration of a polymeric matrix during the processing of sample preparation. In the same way, the use of ruthenium red, a carbohydrate-binding dye, showed that *L. monocytogenes* bind this dye (Borucki et al., 2003; Zameer et al., 2010), which is consistent with the presence of exopolysaccharides. Nevertheless, as this dye can also bind carbohydrates on the bacterial cell surface unrelated to exopolysaccharides (peptidoglycans, teichoic acids, etc), these observations are not conclusive. Finally, it may be generally accepted that *L. monocytogenes* is a poor exopolysaccharide-producer by comparison with other bacterial species and that the putative presence of an extracellular matrix is dependent on the strain and environmental conditions (Borucki et al., 2003; Marsh et al., 2003; Zameer et al., 2010). Recently, Chen et al. (2014) identified a new c-di-GMP-inducible exopolysaccharide which caused cell aggregation in minimal medium and also impaired bacterial migration in semi-solid agar. However, this polysaccharide was not found to promote biofilm formation on abiotic surfaces. Harmsen et al. (2010) showed that in *L. monocytogenes* eDNA may be the only central component of the biofilm matrix and that it is necessary for both initial attachment and early biofilm formation. DNase I treatment resulted in dispersal of biofilms, not only in microtiter tray assays but also in flow cell biofilm assays. A dispersal of pre-existing *L. monocytogenes* biofilms by DNase treatment has also been shown by Nguyen and Burrows (2014). In agreement, the disruption of a putative DNA translocase gene impaired biofilm formation of *L. monocytogenes* on abiotic surfaces (Chang et al., 2013).

### Interspecies Interactions

In food industry settings, interactions of *L. monocytogenes* in multi-species communities are likely, with the bacterial flora in food processing plants to include bacteria with the potential to increase or decrease colonization and biofilm formation by *L. monocytogenes* (Fox et al., 2014). Strains providing enhanced colonization and biofilm formation could contribute to *L. monocytogenes* persistence in food industry premises and consist a potential food safety risk. Bacteria with such properties, e.g., *Pseudomonas* spp., are commonly found on food processing surfaces even after cleaning and disinfection. They are also found to be significant biofilm producers. For instance, Hassan et al. (2004) showed increased attachment of *L. monocytogenes* on wet surfaces pre-colonized with *P. putida*. The change in surface properties due to *P. putida* EPS production which enhanced attachment of *L. monocytogenes* was the most likely cause to explain increased biofilm formation. Other spoilage organisms including *Flavobacterium* sp. have been reported to enhance biofilm formation of *L. monocytogenes* (Bremer et al., 2001). In contrast, biofilm formation by *L. monocytogenes* was reduced in co-cultures with *Pseudomonas fragi* (Norwood and Gilmour, 2001). Co-cultures with various Gram-negatives including *Serratia* spp., *Aeromonas* sp., and *P. fluorescens* have shown similar reductions (Gudmundsdottir et al., 2005; Daneshvar Alavi and Truelstrup Hansen, 2013). In less humid environments, staphylococci and other Gram-positives are regularly present. Co-culture biofilms with *L. monocytogenes* and *S. aureus* showed strain dependence and either increased, decreased or no effect on *L. monocytogenes* biofilm formation (Rieu et al., 2008b). The stimulated biofilm formation seemed to be caused by a *S. aureus*-excreted peptide. Biofilm formation of *L. monocytogenes* was decreased in the presence of *S. sciuri* where nutrient competition and extracellular substances produced by *S. sciuiri* explained the decreased adhesion and biofilm formation (Leriche and Carpentier, 2000). In another relevant study, Weiler et al. (2013) analyzed whether different *L. monocytogenes* strains are interacting with the microbial community of raw milk in terms of biofilm formation and found that the addition of individual *L. monocytogenes* strains to raw milk caused significant shifts in the biofilm biomass, in the chemical, as well as in the bacterial community composition. However, the added *L. monocytogenes* strains were not abundant, since mainly members of the genera *Citrobacter* and *Lactococcus* dominated the mixed culture bacterial biofilm community. In a study of 29 Gram-negative and Gram-positive isolates from food processing plants, 13% of the strains increased *L. monocytogenes* counts in the biofilms, while 53 and 34% had a negative and no effect on *L. monocytogenes* populations, respectively (Carpentier and Chassaing, 2004). Almeida et al. (2011) employed peptide nucleic acid fluorescence *in situ* hybridization (PNA FISH) to characterize *S. enterica*/*L. monocytogenes*/*E. coli* tri-species biofilm and identified two well-defined layers: the top one with *E. coli*, and the bottom one with mixed regions of *L. monocytogenes* and *S. enterica*. The higher growth rate and exopolymer production ability of *E. coli* probably led this microorganism to outcompete the other two. The competition of *L. monocytogenes* serotype 1/2a and 4b strains in mixed-culture biofilms has been demonstrated by Pan et al. (2009), with the serotype 1/2a strains tested to be generally more efficient at forming biofilms and to predominate in the mixed-culture biofilms.

Bacterial biofilms formed by useful technological bacteria, such as lactic acid bacteria (LAB) have the potential to control the development of *L. monocytogenes* through competitive exclusion and the synthesis of organic acids and bacteriocins (Guillier et al., 2008; Minei et al., 2008; Habimana et al., 2009, 2011; Woo and Ahn, 2013; Perez Ibarreche et al., 2014). For instance, Zhao et al. (2004) found 2 out of 413 microbial isolates from drains in food processing facilities to have significant anti-listerial activities. Follow-up studies have shown the potential of these isolates (*Enterococcus durans* and *Lactococcus lactis*) to control and eliminate *L. monocytogenes* from drains in the meat and poultry processing industry (Zhao et al., 2006, 2013). Although the mechanisms are not clear, both strains produce anti-listerial metabolites. Other studies have revealed nutrient competition as the principal mechanism behind the inhibition of *L. monocytogenes* in presence of multi-species biofilm microflora where *L. monocytogenes* often represent a minor part of the biofilm bacterial population (Chorianopoulos et al., 2008; Guillier et al., 2008), although a range of many other growth associated parameters may affect this distribution.

Once adhered to surfaces, *L. monocytogenes* often show enhanced survival and tolerance to food associated stresses which may even be enhanced by biofilm formation (Chavant et al., 2004; Pan et al., 2006; Carpentier and Cerf, 2011; Yun et al., 2012). Tolerance of *L. monocytogenes* to sanitizers and disinfectants has been reported to increase in dual culture biofilms as shown with *L. monocytogenes* and *Lb. plantarum* dual biofilms (van der Veen and Abee, 2011). Other studies have reported increased disinfectant tolerance of *L. monocytogenes* in mixed species biofilms. However, the protecting effects of the secondary species have been difficult to judge due to no results provided on single species biofilm resistance (Fatemi and Frank, 1999; Norwood and Gilmour, 2000). In dual culture biofilms of *L. monocytogenes* and *S. enterica*, the interspecies interactions did not influence either the biofilm forming ability or the resistance of each species to commonly used disinfectants. However, the intra- and interspecies interactions encountered in the biofilms had effect on the population dynamics and the resistance pattern of each *L. monocytogenes* strain present in the biofilm (Kostaki et al., 2012). Interactions between *L. monocytogenes* and *P. putida* provided increased tolerance to *P. putida* biofilm cells to benzalkonium chloride while *L. monocytogenes* tolerance remained unaffected in dual- or mono-species biofilms (Giaouris et al., 2013). This contrast to the study of Saa Ibusquiza et al. (2012) where increased tolerance of *L. monocytogenes* in dual *L. monocytogenes*/*P. putida* biofilms was reported.

Cell-to-cell signaling systems based on QS appear to be involved in bacterial biofilm formation although their exact role is still awaited (Li and Tian, 2012). In *L. monocytogenes*, there are two QS systems: the AI-2 signal system reported in both Gram-negative and Gram-positive bacteria and proposed as a universal, interspecies communication system, and the peptide-mediated QS system Agr, characteristic for Gram-positive bacteria (Waters and Bassler, 2005). Another communication system present in Gram-positive bacteria, involved in the development of competence in *Bacillus subtilis*, is associated with the autoinducer ComX. However, the main genes of this system are absent in the genome of *L. monocytogenes* for which, moreover, natural genetic transformation has never been observed (Borezee et al., 2000). Although *L. monocytogenes* can produce AI-2, experimental evidence indicates that *L. monocytogenes* lack receptors of AI-2. Thus, this suggests that AI-2 is not a communication signal in *L. monocytogenes* (Challan Belval et al., 2006; Garmyn et al., 2009). Concerning the *agr* system described in *L. monocytogenes*, it appears that this regulates major adaptive responses, such as the promotion of biofilm formation, expression of adhesion factors and internalins (Rieu et al., 2007, 2008a; Riedel et al., 2009). It has been shown that *agrA* or *agrD* mutant strains, two of the four genes composing the *agr* locus, were affected in adhesion and the first stage of biofilm formation. However, the *agrA* or *agrD* gene products, a response regulator of a two-component system and a precursor peptide respectively, are parts of a complex signaling system probably involved in multiple physiological regulation. Thus, there is no demonstration to date of the mechanism by which they intervene in biofilm formation.

### Intercellular Interactions in Biofilms of *L. monocytogenes*: Current Knowledge and Concepts for Future Research

*Listeria monocytogenes* is considered as an environmental pathogen because it is capable of saprophytic life in the outside environment while also maintaining the ability to invade and replicate within mammalian cells (Xayarath and Freitag, 2012). This pathogen is capable of forming biofilms which considerably increase its resistance to harsh physicochemical conditions and particularly to cleaning and disinfection treatments. Thus, in this sessile mode of growth, bacteria may persist in production lines and constitute a permanent risk of contamination of food products. Literature has shown that *L. monocytogenes* biofilm formation, maturation, and structure depends on a multitude of external and internal factors, where both intra- and inter-species interactions seem to play an important role (Renier et al., 2011). However, their exact and respective underlying mechanisms are not always characterized, emphasizing that much remains to be investigated. Remarkably, *L. monocytogenes* may display increased disinfection tolerance when this forms biofilms with other species. This is certainly something which is worth to be further studied. Besides such an increase in disinfection resistance, the influence of various biofilm forming conditions on the virulence properties of these bacteria is still largely unexplored. Future research on this area should include both mono and multi-species biofilms and focus on the underlying molecular mechanisms hidden behind any observation. Finally, while peptide sensing is able to promote *L. monocytogenes* biofilm formation (Rieu et al., 2007, 2008a; Riedel et al., 2009), how this is achieved is not yet recognized.

## Escherichia coli

*Escherichia coli* is primarily a commensal species which however also contains important pathogenic strains (Kaper et al., 2004), with the foodborne pathogenic *E. coli* to be diarrheagenic strains (Nataro and Kaper, 1998; Croxen et al., 2013). Those intestinal pathogenic *E. coli* (InPEC) can be subdivided into the following pathotypes, namely ETEC (enterotoxigenic *E. coli*), EAEC (enteroaggregative *E. coli*), DAEC (diffusively adherent *E. coli*), EIEC (enteroinvasive *E. coli*), EPEC (enteropathogenic *E. coli*), and EHEC (enterohemorrhagic *E. coli*), with the recently described subgroup of EAHEC (enteroaggregative and haemorrhagic *E. coli*, Brzuszkiewicz et al., 2011). By definition, EHEC are clinical strains of InPEC and belong to the larger group of Shiga toxin producing *E. coli* (STEC), which pathogenesis is not ascertained.

### Intraspecies Interactions

Various cell surface molecules and structures have been implicated in biofilm formation in *E. coli* (Van Houdt and Michiels, 2005). In this bacterium, a major cell–cell interaction involves the autotransporter Ag43 (Antigen 43; van der Woude and Henderson, 2008), a protein of the Type Va secretion system (T5aSS; Desvaux et al., 2004b). This protein is found encoded in a majority of *E. coli* genomes, from domesticated K-12, commensal to pathogenic strains (van der Woude and Henderson, 2008). While Ag43 was known to be a self-recognizing protein promoting autoaggregation and consequently biofilm formation (Danese et al., 2000; Klemm et al., 2004), it was recently uncovered that its functional domain displays a twisted L-shaped β-helical structure firmly stabilized by a 3D hydrogen-bonded scaffold which facilitates self-association and cell aggregation via a mechanism described as a Velcro-like handshake (Heras et al., 2014). Very interestingly, the expression of the Ag43 is subjected to phase variation (Diderichsen, 1980; Henderson et al., 1997, 1999; Henderson and Owen, 1999). It was recently demonstrated that biofilm formation did not influence the frequency of switch between bacterial cells expressing the Ag43 (phase ON) and those which do not expressed it (phase OFF; Chauhan et al., 2013). Because of the autoaggregation phenotype, the phase ON *E. coli* cells were physically selected and prominent within the biofilm under dynamic flow conditions.

Of note, several other autotransporters can also be involved in cell aggregation, namely AIDA (Sherlock et al., 2004; Girard et al., 2010) and TibA (Sherlock et al., 2005), i.e., SAATs (self-associating autotransporters; Klemm et al., 2006), but also EhaA (Wells et al., 2008), at least when overexpressed. In *E. coli* O157:H7, Cah is a calcium-binding and heat-extractable autotransporter protein homologous to Ag43 and AIDA (Torres et al., 2002). Besides, TolC was supposedly also involved in *E. coli* aggregation (Imuta et al., 2008), as well as curli and Type 1 pili (Barnhart and Chapman, 2006; Tree et al., 2007; Ulett et al., 2007). Expression of Type 1 pili and polar localization of Ag43 can further influence bacterial cell chain formation in biofilm (Vejborg and Klemm, 2009). Within the T5SS (Desvaux et al., 2004a; Henderson et al., 2004), some proteins of the subfamily b (T5bSS) of the two-partner secretion (TPS) are involved in contact-dependent inhibition (CDI; Hayes et al., 2014). This system allows regulating bacterial growth in response to changing environmental conditions upon direct cell–cell interactions (Aoki et al., 2005; Hayes and Low, 2009; Hayes et al., 2010). The CDI activity is very specific since it is limited to target cells of the same species. So far, the role of CDI in the course of biofilm formation has not been investigated in the scientific literature.

Pili are cell-surface supramolecular protein complexes involved in cell-to-cell interactions (Lasaro et al., 2009; Hernandes et al., 2013), which have also been termed fimbriae or curli based on some morphological differences. Curli production occurs via the Type VIII secretion system and is dependent on the CsgD transcription activator, which also promotes cellulose biosynthesis (Brombacher et al., 2006). In *E. coli*, the expression of the curli-specific genes (*csg*) which are clustered in the *csgBA* and *csgDEFG* operons is dependent on a combination of environmental parameters such as low growth temperature and low osmolarity (Hammar et al., 1995; Römling et al., 1998). Previous studies revealed a positive correlation between curli expression, exopolysaccharides production (such as cellulose), and autoaggregation by *E. coli* (Ryu et al., 2004a,b; Uhlich et al., 2006; Tree et al., 2007; Gualdi et al., 2008; Saldaña et al., 2009; Goulter et al., 2010). The *E. coli* common pilus (ECP) represents a remarkable family of extracellular fibers and plays a dual role in early-stage biofilm development and host cell recognition (Garnett et al., 2012). The microcolony formation on biotic surfaces in EPEC is mediated by several adhesins including the type IV bundle-forming pilus (BFP) and the EspA filament, which are also involved in bacterial aggregation during biofilm formation on abiotic surfaces together with the type 1 fimbriae and the antigen Ag43 (Moreira et al., 2006). EAEC forms thick biofilms on the intestinal mucosa by virtue of a plasmid-encoded fimbrial adhesin designated aggregative adherence fimbria I (AAF/I; Czeczulin et al., 1997). EHEC factor for adherence Efa1 confers haemagglutination, adherence to epithelial cells and autoaggregation (Nicholls et al., 2000). Recently, Gómez-Gómez and Amils (2014) characterized a novel *E. coli* sessile behavior termed “crowning” which is developed independently of the adhesiveness of the major components of *E. coli*’s EPS matrix, the function of chemotaxis sensory system, type 1 pili and the biofilm master regulator CsgD, but its formation is suppressed by flagella-driven motility and glucose.

The F episome encodes a Type IVb secretion system (T4bSS) responsible for bacterial conjugation, the so-called sexual pili (Chagnot et al., 2013). It was elegantly demonstrated that the F episome in *E. coli* was not only involved in the horizontal transfer of genetic information to F-recipient cells but actively contributed to biofilm formation (Ghigo, 2001). Conjugation further decreased motility, which increased the biofilm formation in *E. coli* (Barrios et al., 2006; Reisner et al., 2006). As promoters of bacterial colonization and development of mature biofilms by providing aggregative properties, the conjugative plasmids in general should be more systematically considered as a risk factor among foodborne pathogenic bacteria (Dudley et al., 2006; Mliji el et al., 2007; May and Okabe, 2008; Norman et al., 2008); potentially, they cannot only promote intra- but also inter-species genetic transfer even between Gram-positive and Gram-negative bacteria (Goessweiner-Mohr et al., 2013).

Zhang et al. (2007) demonstrated, using DNA microarrays, that the expression of *ycfR*, which encodes the putative outer membrane protein YcfR (BhsA), is significantly induced in *E. coli* biofilms. This protein seems to be involved in the regulation of biofilm formation by decreasing cell aggregation and cell surface adhesion, by influencing the concentration of signal molecules, and by interfering with stress responses. Critical to the development of a biofilm is the elaboration of exo-polysaccharide that contributes to substrate and intercellular adhesion. Indeed, one way to identify the environmental cues that cause a given bacterial species to switch to the biofilm mode of growth is to monitor exo-polysaccharide elaboration *in vitro* (Jefferson and Cerca, 2006). Lipopolysaccharide (LPS) is the major component of the surface of Gram-negative bacteria and its polysaccharide portion is situated at the outermost region. Mutations known to affect the composition of *E. coli* LPS core oligosaccharide affected the biofilm formation which was associated with eDNA (Nakao et al., 2012). The spatial periodicity of *E. coli* cells within a biofilm has been associated to the secretion of the polysaccharide adhesin β-1,6-*N*-acetyl-d-glucosamine PGA (Agladze et al., 2005). Colanic acid also contributes to the biofilm architecture and allows for the formation of voluminous biofilms (Prigent-Combaret et al., 2000).

Quorum sensing has been shown to play a significant role on the surface chemistry and electrokinetic properties of *E. coli* cells, possibly through the regulation of outer membrane macromolecules (Eboigbodin et al., 2006). In addition, *E. coli* cells cultivated with an additional supplement of glucose, displayed a higher concentration of bacterial surface functional groups and a variation in outer membrane proteins, which consequently reduced the tendency for cell-to-cell attachment (Eboigbodin et al., 2007). AI-2 takes part both in intra- and inter-species interactions in *E. coli* and is involved in multiple physiological processes, including biofilm formation, exopolysaccharides production, and determination of cell surface properties via the regulation of the genes encoding outer membrane proteins and putative adhesins (DeLisa et al., 2001). The interactions between *E. coli* cell clusters involving particularly the AI-2 mediated cell-to-cell signaling have been found to play an important role in the spatial organization of the cell clusters in biofilms of *E. coli* (Gu et al., 2013). The two component signal-transduction system Cpx is also believed to act as a strategic signaling pathway for confronting adverse conditions and for settling biofilm communities by activating genes encoding periplasmic-protein-folding and degrading factors (Dorel et al., 2006). Interestingly, in recent years, plant phytochemicals have appeared as a novel promising strategy for controlling biofilm formation and virulence in *E. coli* and other pathogens through interfering with the bacterial cell–cell signaling pathways and the expression of cell surface adhesins (Vikram et al., 2010a,b; Lee et al., 2014a).

### Interspecies Interactions

*Escherichia coli* interacts with other microorganisms and is able to form multi-species biofilms with many of the most common bacterial genera occurring on food processing surfaces including both Gram negatives (e.g., *Pseudomonas, Acinetobacter*) and Gram positives (e.g., staphylococci, *Bacillus*, Castonguay et al., 2006; Marouani-Gadri et al., 2009; Habimana et al., 2010a; Kuznetsova et al., 2013; Liu et al., 2014). Strains isolated from water and food processing environments have shown to stimulate co-adhesion and biofilm formation of *E. coli*. For instance, Castonguay et al. (2006) showed that biofilm formation of non-adherent *E. coli* from drinking water reservoirs was stimulated in dual and other multiculture biofilms with biofilm proficient bacteria from the same environment. The mechanism of biofilm stimulation required direct cell-to-cell contact. All but one of 20 randomly collected bacterial isolates obtained after cleaning and disinfection from a beef processing plant increased the counts of adhered *E. coli* O157:H7 in dual-culture biofilms (Marouani-Gadri et al., 2009). Low-nutrient conditions conferred by growth of the resident strains and which favored *E. coli* biofilm formation was hypothesized as an explanation of the observed phenomenon. Microflora isolates capable of producing biofilms are widely distributed in fresh produce processing facilities, while the presence of persistent biofilm forming strains is also reported (Van Houdt et al., 2004; Liu et al., 2013). Strong biofilm forming plant-associated bacteria promoted the incorporation of *E. coli* O157:H7 in biofilms at 30°C (Liu et al., 2014). Carter et al. (2012) examined the interaction of *E. coli* O157:H7 with spinach leaf indigenous microorganisms during co-colonization and establishment of a mixed biofilm on a stainless steel surface, by using a metagenomics analysis, and revealed competition for essential macronutrients as the primary interaction. Under flow conditions adhesion to glass surfaces of *Acinetobacter calcoaceticus* (Habimana et al., 2010a) or *P. aeruginosa* (Klayman et al., 2009) stimulated adhesion of *E. coli* O157:H7 which was not able to form biofilm in monoculture under dynamic flow conditions. Although the detailed mechanisms are not known, it is likely that the early colonizer provide surface structures or surfactants promoting co-adhesion of *E. coli*. Cell-to-cell dependent interactions promoting retention of *E. coli* O157:H7 in biofilms have also been shown by others (Uhlich et al., 2010). Bacteria affecting biofilm formation of *E. coli* through secreted metabolites have also been reported (Cabellos-Avelar et al., 2006; Lopes et al., 2011; Vacheva et al., 2012).

The above studies show that bacterial interactions can promote pathogenic *E. coli* biofilm formation and even enable adherence deficient strains to form biofilms. This is of significant relevance and highlights that the control of environmental bacteria promoting adherence and biofilm formation of other bacteria can be an important measure to prevent establishment of pathogenic *E. coli* and other pathogens in food processing environments. On the other hand, Da Re et al. (2013) analyzed genetic responses induced in commensal *E. coli* upon entry of a diarrheagenic EAEC or an unrelated *K. pneumoniae* pathogen into a biofilm community and identified some genes involved in limiting colonization of incoming pathogens within commensal biofilm. Valle et al. (2006) demonstrated that all *E. coli* expressing group II capsules release into their environment a soluble polysaccharide that induces physicochemical surface alterations, which prevent biofilm formation by a wide range of Gram positive and Gram negative bacteria. Similarly, Rendueles et al. (2011) identified an *E. coli* biofilm-associated anti-adhesion polysaccharide which reduced susceptibility to invasion and provided rapid exclusion of *S. aureus* from mixed *E. coli* and *S. aureus* biofilms. These last findings identify bacterial interference via surface active compounds produced during competitive interactions as a new strategy to limit biofilm formation on surfaces.

As previously mentioned in the *Salmonella* part of this review, dual cultures of *E. coli* O157:H7 and *S.* Typhimurium showed that EPS-producing strains were able to establish themselves in mixed biofilms more efficiently but also enhanced *E. coli* O157:H7 biofilm formation (Wang et al., 2013). The protective role of an EPS producing strain of *S.* Typhimurium in providing increased resistance to a non-EPS producing *E. coli* O157:H7 strain toward two industrial sanitizers was also shown (Wang et al., 2013). This indicates that EPS-producing strains also may provide protection to sensitive companion strains in mixed species biofilms. *E. coli* and its LPS has been shown to modulate *in vitro* biofilm formation by *Candida* species (Bandara et al., 2009). Putative F pili expressed by EAEC strains boosted mixed biofilm formation when in the presence of aggregative *Citrobacter freundii* (Pereira et al., 2010). *E. coli* transformed with green fluorescent protein (GFP) and *Serratia marcescens* stably co-existed in biofilms but did not affect the growth of each other (Skillman et al., 1998).

Another interesting concern is the correlation between the biofilm forming ability of pathogenic *E. coli* and the presence of probiotic bacteria (Chapman et al., 2012, 2014). Decreased *E. coli* O157:H7 biofilm formation was observed in the presence of EPS produced by a probiotic *Lb. acidophilus* strain by interfering with the expression of *E. coli* surface adhesins (Kim et al., 2009). In the investigations conducted by Kim et al. (2012) cell extract of *Bifidobacterium longum* caused 36% reduction in biofilm formation by *E. coli* O157:H7. Significant inhibition in AI-2 QS activity in *E. coli* O157:H7 was also observed, while proteome analysis showed that seven proteins were differentially regulated in *E. coli* in the presence of *Bifidobacterium* cell extract. However, Miyazaki et al. (2010) observed no inhibitory effect of the culture supernatant of *Bifidobacterium* spp. against biofilm formation by EAEC. Andrzejewska and Sobieszczanska (2013) showed that *Lb. casei* inhibited the biofilm formation by EAEC. The reason for this was not explained. Obviously probiotic bacteria may reduce biofilm formation by *E. coli*, but further analysis is still required.

Interspecies interactions in multi-species biofilms also include HGT (Madsen et al., 2012; Van Meervenne et al., 2014). Conjugative transfer of a plasmid encoding a type 3 fimbriae rendered a non-biofilm producing uropathogenic *E. coli* strain to be a significant member in a mixed biofilm (Ong et al., 2009). Conjugative transmission of natural plasmids carried by the *E. coli* lead to biofilm expansion in mixed *E. coli* biofilms (Reisner et al., 2006). Another study showed HGT of shiga toxin encoding genes (*stx*) to occur by transduction in *E. coli* (Solheim et al., 2013). BdcA binds the ubiquitous bacterial signal c-di-GMP and has been found to control biofilm dispersal in *P. aeruginosa* and *Rhizobium meliloti* via conjugation from *E. coli* in mixed-species biofilms (Ma et al., 2011). These reports illustrate the potential of HGT to spread determinants involved in biofilm formation, dispersal and virulence which have relevance for food-associated *E. coli*. Surely, the impact of the transfer of conjugative plasmids on multi-species biofilm formation is dependent on both the type of the plasmid and the plasmid host (Røder et al., 2013).

So far, only a few studies indicated the important role of AI-2 QS system in biofilm formation by *E. coli* (González Barrios et al., 2006; Silagyi et al., 2009; Zhou et al., 2014). González Barrios et al. (2006) showed that the addition of AI-2 enhanced biofilm formation by *E. coli* by stimulating flagellar motion and motility. Silagyi et al. (2009) observed that *E. coli* O157:H7 produced maximum levels of AI-2 signals in 12 h of incubation in tested foods and next formed strong biofilm in 24 h of incubation. In other studies AI-2-based QS activity of *E. coli* O157 did not affect biofilm formation in monocultures (Yoon and Sofos, 2008b). Indole has been proved as an interspecies signal that decreases *E. coli* biofilms through SdiA and increases those of pseudomonads (Lee et al., 2007). *E. coli* also possess receptors for AHL which can be produced by other bacteria (Moons et al., 2006; Ren et al., 2013). The potential effect of AHL producing bacteria on *E. coli* biofilm formation should be studied in multiculture biofilms.

### Intercellular Interactions in Biofilms of *E. coli*: Current Knowledge and Concepts for Future Research

*Escherichia coli* possesses a wealth of cell surface structures involved in intercellular interactions during biofilm development (mainly by provoking cell aggregation). Their respective contribution and regulation in the course of sessile growth still remain to be further elucidated at a global scale respective to crucial environmental conditions as a function of the pH, temperature, and/or available nutrients. Of note for instance, the mechanism, regulation and function of Ag43 are quite restricted to studies using the non-pathogenic and domesticated *E. coli* K12 strain but further investigations respective to the different subfamilies of Ag43 and pathogenic *E. coli* species are undoubtedly required (van der Woude and Henderson, 2008). As another example and in addition to bacterial growth inhibition, CDI system might have other but as yet uncovered physiological functions; its potential involvement in the course of sessile development is particularly relevant and intriguing, which would hopefully trigger intense research investigations in the near future. Worth mentioning is also the growing evidence that biofilms of *E. coli* and other microorganisms represent an ideal microenvironment for HGT. Natural conjugative plasmids have been shown to promote the development of mature *E. coli* biofilms by providing aggregative properties, promoting cell-surface interactions, and stimulating colanic acid and curli production (Ghigo, 2001). However, there is no data in about the HGT by transformation (described as uptake of free DNA from the surrounding environment) in multi-species biofilms formed with *E. coli* contribution. For foodborne pathogenic *E. coli* like EHEC, their extremely low infectious dose combined with their ability to form biofilms and associate in multi-species biofilms poses an extra challenge. Finally, the possible effect of AHLs produced by other food related bacteria on *E. coli* biofilm formation is for sure another intriguing case of future research.

## Staphylococcus aureus

*Staphylococcus aureus* is a Gram-positive, ubiquitous bacterial species commonly found on the skin and hair, as well as in the noses and throats of people and animals. It is the causative agent of a wide spectrum of human infections (Otto, 2013) and is also often responsible for foodborne intoxications through the production of heat stable enterotoxins in a variety of food products (Hennekinne et al., 2012). *S. aureus* can produce a multilayered biofilm embedded within a glycocalyx or slime layer with heterogeneous protein expression throughout (O’Gara, 2007; Archer et al., 2011; Periasamy et al., 2012; Laverty et al., 2013). Especially *S. aureus* harbors a variety of proteinaceous and non-proteinaceous adhesins that mediate attachment to a multitude of host factors, such as extracellular matrix and plasma proteins and human host cells, or intercellular adhesion, which is essential for biofilm accumulation (Heilmann, 2011).

### Intraspecies Interactions

The major constituents of staphylococcal biofilms are polysaccharides, such as the polysaccharide intercellular adhesin (PIA) or poly-*N*-acetyl glucosamine (PNAG), cell surface and secreted bacterial proteins, and eDNA (Izano et al., 2008). However, the exact composition of biofilms often varies considerably between different strains of staphylococci and between different sites of infection by the same strain. PIA is encoded by the *icaADBC* operon, yet details of its biosynthesis are limited (Atkin et al., 2014). Regulation of *icaADBC* is extremely complex, this is influenced by many environmental factors and involves an array of coordinated regulatory mechanisms which have yet to be fully elucidated (Mack et al., 2004; O’Gara, 2007; Cue et al., 2012). Biofilm-specific transcriptional regulators include Rbf, which mediates the induction of biofilm formation at the cell–cell interaction stage in response to glucose and osmotic stress (Lim et al., 2004; Cue et al., 2009), and IcaR and TcaR, both of which negatively regulate biofilm formation (Jefferson et al., 2004). Global transcriptional regulators include staphylococcal accessory regulator (SarA), which is required for biofilm formation (Valle et al., 2003; Trotonda et al., 2005; Sambanthamoorthy et al., 2008; Mrak et al., 2012), and the two-component regulator ArlRS, a repressor of biofilm formation (Toledo-Arana et al., 2005). Inactivation of a global regulator of the bacterial stress response, the alternative transcription factor sigma(B), resulted in a biofilm-negative phenotype and loss of salt-induced biofilm production (Rachid et al., 2000). However, Valle et al. (2003) demonstrated that complete deletion of sigmaB did not affect PIA/PNAG production and biofilm formation, although it slightly decreased *ica* operon transcription. You et al. (2014) identified a new operon, *gbaAB* (glucose induced biofilm accessory gene) that is involved in the regulation of the multicellular aggregation step of *S. aureus* biofilm formation in response to glucose and showed that this regulation may be mediated through the *ica* operon. Osmotic stress was found to induce biofilm formation in a *S. aureus* mucosal isolate (Rachid et al., 2000).

Undoubtedly, the genetic and molecular basis of biofilm formation in staphylococci is multifaceted (Götz, 2002). Beyond PIA/PNAG, evidence is now emerging for the existence of *ica*-independent biofilm mechanisms capable of mediating intercellular accumulation in both *S. aureus* and *S. epidermidis* (O’Gara, 2007). Thus, a number of biofilm-negative mutants have been isolated in which PIA production appears to be unaffected. Two of the characterized mutants were affected in the major autolysin (*atlE*, Biswas et al., 2006; Bose et al., 2012) and in D-alanine esterification of teichoic acids (*dltA*, Gross et al., 2001). Teichoic acids are anchored to the outer layer of the cytoplasmic membrane via a glycolipid (lipoteichoic acid, LTA) or covalently to the cell-wall peptidoglycan (wall teichoic acid, WTA). A *S. aureus ypfP* mutant with strongly reduced LTA content was completely unable to form biofilm on plastic (Fedtke et al., 2007). WTA-deficient *S. aureus* mutants showed a higher degree of cell aggregation, but had reduced initial adherence to abiotic surfaces and had a reduced capacity to form biofilms under both steady-state and flow conditions (Vergara-Irigaray et al., 2008). eDNA provides structure and stability in mature biofilms of *S. aureus* (Izano et al., 2008), and many other species (Das et al., 2013b). Secreted proteins may also influence *S. aureus* multicellular behavior. Caiazza and O’Toole (2003) showed that alpha-toxin (also known as α-hemolysin or Hla), a secreted, multimeric, hemolytic toxin encoded by the *hla* gene, plays an integral role in *S. aureus* biofilm formation and is required for cell-to-cell interactions. Similarly, Anderson et al. (2012) showed that alpha-toxin also promotes *S. aureus* mucosal biofilm formation.

The surface of *S. aureus* is “decorated” with proteins that are in most cases covalently anchored to the cell wall peptidoglycan via an LPxTG motif cleaved by sortase A (SrtA). Interestingly, the overexpression of SrtA has resulted in increased levels of biofilm formation in some *S. aureus* strains (Sibbald et al., 2012). Structural and functional analysis has identified four distinct classes of surface proteins in this bacterium, of which microbial surface component recognizing adhesive matrix molecules (MSCRAMMs) are the largest class. These surface proteins have numerous functions, including adhesion to and invasion of host cells and tissues, evasion of immune responses and biofilm formation (Foster et al., 2014; Speziale et al., 2014). Surface proteins Bap (biofilm-associated protein), Eap (extracellular adherence protein), or PSM (phenol soluble modulin) promote *S. aureus* adherence to host cells and surfaces, as well as cell aggregation (Cucarella et al., 2001; Hussain et al., 2008; Thompson et al., 2010; Schwartz et al., 2012). Interestingly, Arrizubieta et al. (2004) found that Bap binds Ca^2+^ with low affinity and that this binding renders the protein non-competent for biofilm formation and for intercellular adhesion, while Tormo et al. (2007) described a process of phase variation that affects the expression of Bap in *S. aureus*. Thus, it is possible that *S. aureus* can detach from a biofilm by switching to a Bap-negative state. Schwartz et al. (2012) showed that the PSMs’ polymerization and aggregation into amyloid fibers stabilize and promotes *S. aureus* biofilm integrity. Intercellular auto-aggregation is also favored by SasG (*S. aureus* surface protein G; Corrigan et al., 2007; Kuroda et al., 2008; Geoghegan et al., 2010) and SasC (Schroeder et al., 2009), but inhibited by the delta hemolysin (Hld or PSM; Vuong et al., 2003).

Other examples of important surface proteins identified to be involved in *S. aureus* biofilm formation include accumulation-associated protein (Aap), clumping factor A (ClfA), staphylococcal surface protein (SSP1; Foster et al., 2014), protein A (Spa; Merino et al., 2009), serine-aspartate repeat protein SdrC (Barbu et al., 2014), and SraP, a surface-exposed serine-rich repeat glycoprotein (SRRP; Sanchez et al., 2010). Aap and SasG are homologous proteins containing sequence repeats known as G5 domains, which self-associate in the presence of Zn^2+^ resulting in the formation of extensive adhesive contacts between cells (Geoghegan et al., 2010; Conrady et al., 2013). Surface proteins with tandem G5 domains are also found in other bacterial species, suggesting that this mechanism for intercellular adhesion in biofilms may be conserved among staphylococci and other Gram-positive bacteria (Conrady et al., 2008). Expression of SasG masked the ability of exponentially grown *S. aureus* cells expressing protein A (Spa), clumping factor B (ClfB) and the fibronectin binding proteins A and B (FnBPA and FnBPB) to bind to IgG, cytokeratin 10 and fibronectin, respectively (Corrigan et al., 2007). SasG-expressing strains formed peritrichous fibrils of varying density on the cell wall, and also formed biofilm independently of the PIA. It was concluded that the fibrillar nature of SasG explains its ability to mask binding of *S. aureus* MSCRAMMs to their ligands and to promote formation of biofilm. *S. aureus* mutant strains unable to express the FnBPA and FnBPB lacked the ability to adhere to fibronectin and to form biofilms (O’Neill et al., 2008; McCourt et al., 2014). On the contrary, the expression of these two proteins increased bacterial aggregation suggesting that fibronectin-binding proteins can promote the accumulation phase of biofilm. Loss of fibronectin-binding proteins reduced the initial adherence of bacteria, indicating that these proteins are also involved in primary attachment.

Noteworthy, bacterial variants of *S. aureus* called small colony variants (SCVs) originate by mutations in metabolic genes, resulting in emergence of auxotrophic bacterial subpopulations (Melter and Radojevič, 2010). Environmental pressure such as antibiotics, select for isogenic SCV cells that are frequently found coexisting with their parent wild-type strains in a mixed bacterial culture. Such a menadione-auxotrophic *S. aureus* SCV displayed an autoaggregative phenotype and formed highly structured biofilms, consisting of large microcolonies separated by channels, and contained more biomass as well as significantly more PIA than wild-type biofilms (Singh et al., 2010).

Cell population density-dependent regulation of gene expression is an important determinant of bacterial biofilm formation. Staphylococci have two QS systems: the accessory gene regulator (*agr*) which is genus specific and uses a post-translationally modified peptide as an autoinducing signal and *luxS*, which is found in a variety of Gram-positive and Gram-negative bacteria (Kong et al., 2006). Importantly, unlike many QS systems described in Gram-negative bacteria, Agr and LuxS of staphylococci reduce rather than induce biofilm formation and virulence during biofilm-associated infection. Agr enhances biofilm detachment by up-regulation of the expression of detergent-like peptides, whereas LuxS reduces cell-to-cell adhesion by down-regulating expression of biofilm exopolysaccharide via an *icaR*-activation pathway (Yu et al., 2012). However, the role of the Agr QS system appears to vary depending on the strain or growth conditions, as disruption of *agr* can inhibit, enhance, or have no effect on biofilm formation (Yarwood et al., 2004).

### Interspecies Interactions

Studying microbial interactions and adaptive processes leading to co- or poly-bacterial infections is evidently important. *P. aeruginosa* and *S. aureus* are the most prevalent pathogens in airway infections of cystic fibrosis (CF) patients, while significant research has been conducted on how these pathogens coexist and interact with each other (Hoffman et al., 2006; Davies and Marques, 2009; Chew et al., 2014). Interestingly, wild-type *P. aeruginosa* PAO1 has been found to facilitate *S. aureus* microcolony formation when both are grown in co-culture biofilms in a flow-chamber system (Yang et al., 2011a). Further investigations revealed that eDNA behaves as an essential EPS material shared by both species in co-culture biofilms, which facilitates interspecies interactions and that *P. aeruginosa* type IV pili are required for this process, probably through their ability to bind to eDNA. In another recent study, Fugère et al. (2014) found that culture supernatants from 63 *P. aeruginosa* clinical isolates retrieved from CF adult patients triggered a wide range of biofilm-stimulatory activities when added to the culture of a control *S. aureus* strain. However, when studying co-isolated pairs of *P. aeruginosa* and *S. aureus* retrieved from patients showing both pathogens, *P. aeruginosa* supernatants stimulated less biofilm production by the *S. aureus* counterparts compared to that observed using the control *S. aureus* strain. This suggests that colonization of the CF lungs promotes some type of strain selection, or that co-existence requires specific adaptations by either or both pathogens. In the same context, Wollenberg et al. (2014) demonstrated that coproporphyrin III (CIII), a diffusible small molecule excreted by nostril- and skin-associated *Propionibacterium* spp., induces *S. aureus* aggregation in a manner dependent on dose, growth phase, and pH. In another study on a polymicrobial wound infection, the presence of *P. aeruginosa* resulted in induced expression of *S. aureus* virulence factors (Pastar et al., 2013).

Bacteria and fungi are found together in a myriad of environments and particularly in a biofilm, where adherent species interact through diverse mechanisms (Shirtliff et al., 2009). The fungal species *C. albicans* and *S. aureus* are responsible for a majority of hospital-acquired infections and are increasingly co-isolated from implant-associated polymicrobial infections creating an incremental health care problem (Harriott and Noverr, 2009). This polymicrobial biofilm formation and subsequent resistance seems to be a multifactorial process that may require a unique combination of fungal and/or bacterial adhesins (Harriott and Noverr, 2010; Peters et al., 2012b). Thus, synergistic effects between both species seem to facilitate the formation of dual-species biofilms (Millsap et al., 2001). Confocal laser scanning microscopic (CLSM) analyses of such biofilms have revealed a unique biofilm architecture where *S. aureus* is commonly associated with the hyphal elements of *C. albicans* (Peters et al., 2010). In addition, during such co-existence, *S. aureus* is known to present enhanced pathogenesis due to the differential regulation of specific virulence factors (Peters et al., 2010). Interestingly, Fehrmann et al. (2013) also revealed the protective role of *S. aureus* fibrinogen-binding proteins coagulase and Efb against the phagocytosis of *Candida* cells by granulocytes.

Besides synergistic interactions, antagonistic ones have also been observed in mixed culture biofilms of *S. aureus* and other species. Thus, Malic et al. (2011) studied *in vitro* biofilms generated either individually or in dual combinations by *P. aeruginosa, S. aureus, Streptococcus oralis*, and *Micrococcus luteus* and observed distinct species antagonism with apparent antagonism of pathogenic species over “commensal” ones. Iwase et al. (2010) showed that the serine protease Esp secreted by a subset of commensal *S. epidermidis*, inhibits biofilm formation and nasal colonization by *S. aureus* and also destroys pre-existing biofilms of this pathogen. Furthermore, Esp enhances the susceptibility of *S. aureus* in biofilms to immune system components. Similar results were also obtained by Sugimoto et al. (2013) who demonstrated that Esp inhibits *S. aureus* colonization and biofilm formation by degrading specific proteins that are crucial for biofilm construction and host-pathogen interaction. Likewise, Chen et al. (2013) showed that Esp cleaves autolysin (Atl)-derived murein hydrolases and prevents staphylococcal release of DNA, which serves as extracellular matrix in biofilms.

Environmental biofilms grown in seawater on agar containing spent *S. aureus* filtrate were more inhibitory to *S. aureus*, as compared to environmental biofilms grown on plain agar (without added spent *S. aureus* filtrate; Lafleur et al., 2013). Jiang et al. (2011) isolated a bacterial exopolysaccharide (A101) from the culture supernatant of the marine bacterium *Vibrio* sp. QY101 able to inhibit cell aggregates and cell-surface interactions in *S. aureus*. Similarly, Rendueles et al. (2011) also isolated a new type of released high-molecular-weight polysaccharide from *in vitro* mature biofilms formed by natural *E. coli* isolates, whose production reduced susceptibility to invasion and provided rapid exclusion of *S. aureus* from mixed *E. coli* and *S. aureus* biofilms. Sadowska et al. (2010) demonstrated competitive direct interactions due to the activity of antagonistic substances produced by *P. aeruginosa* and *Lb. acidophilus* against planktonic and sessile populations of *S. aureus* strains. Similarly, Walencka et al. (2008) showed the inhibitory activity of surfactants obtained from three *Lb. acidophilus* strains on the ability of *S. aureus* and *S. epidermidis* to form biofilms, while also demonstrated that the addition of surfactants to preformed mature staphylococci biofilms accelerated their dispersal.

**FIGURE 2:**
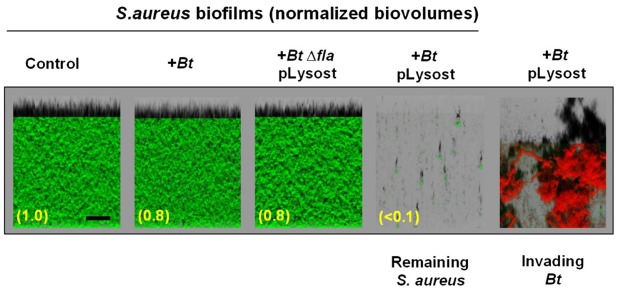
**Biocide-carrying motile ***B. thuringiensis*** eradicates and supplants ***S. aureus*** biofilms.** Confocal observations of *S. aureus* RN4220 GFP biofilms after treatment with motile *B. thuringiensis* 407 (+*Bt*), motile *Bt* expressing lysostaphin (+*Bt* pLysost), and non-motile cells expressing lysostaphin (+*Bt Δfla* pLysost). In these experiments, *S. aureus* biofilms were 24 h old before being exposed to *Bt*. (Scale bar = 30 μm.) In the lower left-hand corner of each confocal image is the quantification of the residual biovolume (μm^3^) of *S. aureus* GFP biofilms after contact with *B. thuringiensis*; each value is the average of ∼18 measurements performed in at least nine independent wells. Figure obtained after permission from Houry et al. (2012).

The interactions in dual species biofilms between *L. monocytogenes* EGD-e and several strains of *S. aureus* have also been studied, with *S. aureus* sessile population to either be increased or decreased or remain unaffected in the presence of *L. monocytogenes* (Rieu et al., 2008b). Respectively, the population of *L. monocytogenes* in dual species biofilms was not affected by the presence of *S. aureus* isolates except for one strain (either when this was *in situ* present or just its supernatant). In an intriguing study, Houry et al. (2012) studied the integrity of biofilms formed by *S. aureus* when this was challenged using either of two motile *Bacillus* species (*B. thuringiensis* and *B. subtilis*) expressing the *S. aureus*-specific cell wall endopeptidase, lysostaphin. Biofilms that were untreated or treated with non-motile *B. thuringiensis* expressing lysostaphin remained essentially intact, and their biovolumes were not statistically different. Remarkably, motile *B. thuringiensis* producing lysostaphin eradicated *S. aureus* within 24 h (Figure 2). Experiments using corresponding *B. subtilis* strains gave results equivalent to those obtained with *B. thuringiensis*. Besides competitive interactions, these results clearly demonstrate that motility gives toxin-carrying bacteria access deep into the biofilm layers to eradicate a preexisting population.

### Intercellular Interactions in Biofilms of *S. aureus*: Current Knowledge and Concepts for Future Research

The matrix in which *S. aureus* cells are encased in a biofilm often consists of the PIA. However, in recent years, many surface proteins capable of promoting biofilm development in the absence of PIA/PNAG have been described. Like in many other species, eDNA also seems to provide a stable architectural organization in *S. aureus* biofilms. While significant progress in elucidating the role of the *icaADBC*-encoded PIA in staphylococcal biofilm development has been made, our understanding of how the *ica* locus and PIA/PNAG biosynthesis are regulated is far from complete and many questions remain. Interestingly, recent results on dual-species *S. aureus* and *P. aeruginosa* biofilm formation have provided insights on bacterial interactions and support the emerging perspective of a co-adaptation and interspecies cooperation that is largely contrasting with studies focusing on the competitive/inhibitory interactions between both bacterial species (Hoffman et al., 2006). Staying in the clinical settings, *S. aureus* and *Candida* species are increasingly co-isolated from implant-associated biofilm infections with amplified virulence during co-infection to occur. However, nothing is yet known on such putative virulence enhancement in *S. aureus* cells forming polymicrobial biofilms in food processing environments. This should be studied since this may significantly compromise food safety. Like in other bacteria, inter-species interactions encountered within *S. aureus* biofilms may be cooperative, antagonistic or even neutral. These largely depend on the bacteria and environmental conditions employed. Special attention should thus be given when someone is trying to formulate general conclusions. Undoubtedly, future studies on multi-species biofilms formed under food relevant conditions will shed light on this fascinating research area. Such information could then serve as a basis for exogenously modulating the interactions between biofilm constituents, resulting in novel approaches for controlling biofilm activities.

## Conclusions and Future Prospects

It is now well accepted that biofilms represent a microbial phenotype, characterized by an explicit organization level, wherein microorganisms are involved in complex intercellular interactions that occur both within and between species and can be either competitive or cooperative (Elias and Banin, 2012; Rendueles and Ghigo, 2012). Such interactions are surely important for the selection of a specific microflora in a given ecological niche (Diaz, 2012; Boon et al., 2014). Moreover, the expression of different cell surface adhesins, their cognate receptors, and exopolymeric components by individual cell types within a biofilm can also contribute to overall biofilm development (Jefferson and Cerca, 2006; Garnett and Matthews, 2012; Das et al., 2013b; Demuyser et al., 2014). Surely, biofilms represent the most frequent mode of growth for many microbes. While headway is being made in understanding their formation and development, we are still far from being able to describe all of these processes from a molecular perspective (Shirtliff et al., 2002). As further insights into this complicated life style are made available, new targets to be exploited will arise, giving us a much wider scope to address problematic biofilms. In this context, intercellular interactions have a profound influence on the formation, structure, and physiology of biofilms (Moons et al., 2009).

Interactions encountered at the stage of microbial adhesion determine the initial community structure (i.e., which species are present) of the developing biofilm. As biofilm accumulation subsequently proceeds, stabilizing interactions between species can lead to increased biofilm thickness and stability and influence biofilm architecture. In addition, physiological interactions between microbial populations increase both the genetic and metabolic flexibility of the community. However, not only the microbial participants but also the environmental conditions in the niche determine the shape and phenotype of a mixed biofilm. Although significant progress in understanding intercellular interactions and their role in microbial growth, survival and virulence have been obtained in recent years, the molecular mechanisms of the regulatory networks involved in sensing and responding to environmental stimuli remain to be elucidated. Moreover, studies on mixed biofilms are beginning to unravel the complexity of interspecies interactions and their impact in clinical, industrial, and environmental settings (Wuertz et al., 2004).

The food industry, authorities and legislation tend to focus on surveillance and control of pathogenic bacteria like *Salmonella* spp., *L. monocytogenes*, STEC, and *S. aureus*. However, the majority of microorganisms in the food processing environment are non-pathogenic. Undoubtedly, the fate of pathogens in sessile multi-species communities may be affected by the type of other bacteria present, thus knowledge of the composition of such communities is important to understand survival and growth of pathogenic bacteria in the food industry (Jahid and Ha, 2014). Thus, the fate of each pathogen in multiculture biofilms including tolerance to food associated stresses clearly depends on the characteristic of co-cultured bacterium. An obvious approach to inhibit the virulence of biofilms would be to prevent the incorporation of potentially pathogenic organisms into biofilms. Since the incorporation of some organisms into biofilms is dependent upon other antecedent biofilm residents, it may be possible to identify such dependencies for specific pathogens and target these antecedent organisms for elimination. Moreover, since many unwanted bacteria are secondary colonizers and adhere on a pre-existing biofilm by means of adhesins, increasing or blocking available binding sites can potentially shift a community toward being less harmful. Shifting nutrients can also be used for directing biofilm functionality (Stoodley et al., 1998). Additionally, hydrodynamic conditions can significantly influence many of the processes involved in biofilm development. However, the effects of these on QS and biofilm formation require further study (Purevdorj et al., 2002).

The properties of biofilms providing protection to environmental stresses and increased resistance to antimicrobial agents are a big challenge (Coenye, 2010; Bridier et al., 2015). Furthermore, resistance is usually enhanced when multiple bacterial species coexist in biofilms. These protective, synergistic effects are highly relevant in many aspects of risk assessment and should be taken into account in selection and evaluation of treatment regimes and cleaning procedures. Bacterial biofilms can endure high concentrations of biocides, and new strategies for controlling them must therefore replace or complement the use of standard disinfectants, for example, by targeting the extracellular matrix to cause dispersal or increased antimicrobial susceptibility. As an example, eDNA is a matrix component of most biofilms, and is therefore an attractive target (Okshevsky and Meyer, 2015). Thus, its enzymatic degradation can prevent, disperse, or sensitize biofilm to antimicrobials (Okshevsky et al., 2014). In addition, given the typical involvement of QS in biofilm development and virulence, QS inhibitors could be used to reduce these characteristics (Burt et al., 2014). These molecules act primarily by quenching the QS system (Defoirdt et al., 2013). In fact, many organisms produce such inhibitors (e.g., halogenated furanones, *N*-acyl homoserine lactonases and acylases; Du et al., 2014), while synthetic compounds have also been successfully developed (de Lima Pimenta et al., 2013). However, despite the capacity of such compounds to influence the formation and virulence of single species biofilms (Rasmussen and Givskov, 2006; Lazar, 2011), their effect on multi-species biofilms so far remains largely unexplored.

Undoubtedly, biofilms should be envisioned as continuously evolving dynamic entities that cannot merely be seen as the sum of all components therein (Yang et al., 2011b; Burmølle et al., 2014). It is now clear that the physiology and function of these complex bacterial communities vary much from that of the individual species when examined as monocultures, and in some cases the underlying mechanisms are known. Accordingly, it follows that insights gained from research based on planktonic cells or even single species biofilms cannot readily be extrapolated to multi-species consortia. Evidently, we are just beginning to understand the complexity of biofilms, but it is already clear from the above examples that much is to be gained in doing so. Different approaches need to be combined, by using high throughput and high resolution methods applied in combination, for better understanding of these complex communities. As an example, comparisons of transcriptomes and proteomes from mono- and multi-species biofilms may allow identification of genes and proteins of which expression is affected by the presence of other species. Therefore, both underlying mechanisms and consequences of co-culturing may be revealed. Continued expansion of such information in the near future will be dependent on the development of new technologies designed to simultaneously identify multiple properties within biofilms. Such advanced knowledge on the physiology of complex biofilms formed by foodborne pathogenic bacteria and the interactions therein will be indispensable for the development of methods for controlling them in food areas.

### Conflict of Interest Statement

The authors declare that the research was conducted in the absence of any commercial or financial relationships that could be construed as a potential conflict of interest.
